# Neonatal Vitamin D Status and Risk of Asthma in Childhood: Results from the D-Tect Study

**DOI:** 10.3390/nu12030842

**Published:** 2020-03-21

**Authors:** Fanney Thorsteinsdottir, Isabel Cardoso, Amélie Keller, Maria Stougaard, Peder Frederiksen, Arieh Sierra Cohen, Ekaterina Maslova, Ramune Jacobsen, Vibeke Backer, Berit Lilienthal Heitmann

**Affiliations:** 1 Research Unit for Dietary Studies, The Parker Institute, Bisbebjerg og Frederiksberg Hospital, Nordre Fasanvej 57, 2000 Frederiksberg, Denmark; 2 Danish Center for Neonatal Screening, Department of Clinical Biochemistry and Immunology, Statens Serum Institute, 2300 Copenhagen, Denmark; 3 Department of Primary Care and Public Health, Imperial College London, London W6 8RP, UK; 4 Centre for Fetal Programming, Department of Epidemiology Research, Statens Serum Institut, 2300 Copenhagen, Denmark; 5 Department of Pharmacy, University of Copenhagen, 2100 Denmark; 6 Center for Physical Activity Research, Rigshospitalet and Copenhagen University, 2100 Copenhagen, Denmark; 7 The Boden Institute of Obesity, Nutrition, Exercise & Eating Disorders, University of Sidney, Sidney, NSW 2006, Australia; 8 The Department of Public Health, Section for General Medicine, University of Copenhagen, 2100 Copenhagen, Denmark

**Keywords:** neonatal, vitamin D, asthma, childhood

## Abstract

Background: low vitamin D status in pregnancy can influence the offspring’s lung function and contribute to childhood asthma development. The objective of this study was to examine the influence of neonatal vitamin D status on the development of asthma among children age 3–9 years in a large population sample. Method: in a case-cohort study utilizing a Danish biobank and register data we examined the association between neonatal 25-hydroxyvitamin D_3_ (25(OH)D_3_) concentrations and incidence of asthma among children aged 3–9 years. Cases of asthma (*n* = 911) were randomly selected among all cases of asthma in the Danish National Patient Register from children born between 1992 and 2002. The sub-cohort (*n* = 1423) was randomly selected among all children born in the same period. We used a weighted Cox proportional hazard model assessing the hazard of first asthma diagnoses by quintiles of 25(OH)D_3_. Results: the median 25(OH)D3 (interquartile range) for asthma cases was 23 nmol/L (14–35) and the sub-cohort 25 nmol/L (14–40). The hazard ratio for developing asthma between ages 3 and 9 years was lower for children in the fifth quintile of neonatal 25(OH)D_3_ compared to children in the first quintile, both in the unadjusted (0.61 95% CI: 0.46–0.80) and adjusted (0.55 95% CI: 0.39–0.77) analyses. Conclusion: the results from our study suggest that higher neonatal vitamin D concentration may reduce the risk of developing childhood asthma at ages 3–9 years, indicating that neonatal vitamin D status as a proxy of vitamin D status during the prenatal period is important for normal immune- and lung development.

## 1. Introduction

Asthma is a chronic respiratory disease, involving both the immune and respiratory system. It often develops as early as in the first months of life and the estimated global prevalence among children 6–7 years old is 9.4% [1]. Such early debut suggests that prenatal environment might play an important role in asthma development [2]. Asthma is a heterogeneous disease with different phenotypes. Early onset childhood asthma and allergic asthma is more prevalent among boys, while incidence in girls tend to increase around puberty [3,4].

Vitamin D is a fat-soluble vitamin and secosteroid hormone, primarily produced in the skin from sun exposure to ultraviolet-B radiation, and possible to obtain from few dietary sources. The fetus is entirely dependent on maternal vitamin D, which is transmitted across the placenta in the form of 25-hydroxyvitamin D (25(OH)D), which is the main circulation metabolite of vitamin D [5]. Vitamin D deficiency is common among otherwise healthy pregnant women [6] and associated with the development of several adverse health outcomes in both women and offspring [7,8,9]. In particular, low gestational vitamin D can influence the offspring’s lung and immune functions and contribute to childhood asthma development [10,11]. 

Joint analysis of two randomized controlled trials (RCTs) [12,13] examining association between prenatal vitamin D supplementation and risk of asthma revealed a significant 26% reduced risk [14]. However, previous observational studies examining prenatal or neonatal 25(OH)D status in relation to the offspring’s asthma risk found no association [15,16,17,18]; this could be due to small sample sizes and/or short follow up. The aim of this study was to examine the influence of neonatal vitamin D status on the development of asthma among children aged 3–9 years in a large population sample. We used a case-cohort study design comparing neonatal 25(OH)D concentrations to test the hypothesis that higher levels of neonatal 25(OH)D are associated with reduced risk of childhood asthma.

## 2. Methods

### 2.1. Data Sources

We identified the study population using the Danish Civil Registration System (DCRS) including all individuals born in Denmark [19]. Individuals in DCRS are registered with a unique 10-digit Central Personal Register (CPR) number that can be used to identify individuals in other Danish registers and databases. Cases of asthma were identified using the Danish National Patient Register (DNPR), a nationwide registry containing information on all hospital admissions, including date of admission and discharge diagnoses, according to the international classification of diseases (ICD) system [20]. 

From May 1981 and onwards, all newborns in Denmark have capillary blood samples taken by heel prick within one week from birth [21]. The samples are collected for routine screening of congenital disorders and stored as dried blood spot samples (DBSSs). After screening, residual DBSSs are stored at the Biological Specimen Bank for Neonatal Screening at Statens Serum Institute (SSI) at −20 °C. Vitamin D status was assessed by measuring 25(OH)D concentrations from DBSSs. Measured 25(OH)D concentrations from DBSSs are highly correlated with cord blood concentrations [22]; measures from DBSSs and serum frozen for 22 and 40 years, respectively, have been shown to be unbiased towards inter-individual variation and deterioration to be minimal [23,24].

Information on covariates was obtained from DCRS [19], the Danish Medical Birth Register [25], DNPR [20], and Statistics Denmark.

### 2.2. Study Population

**Figure 1:**
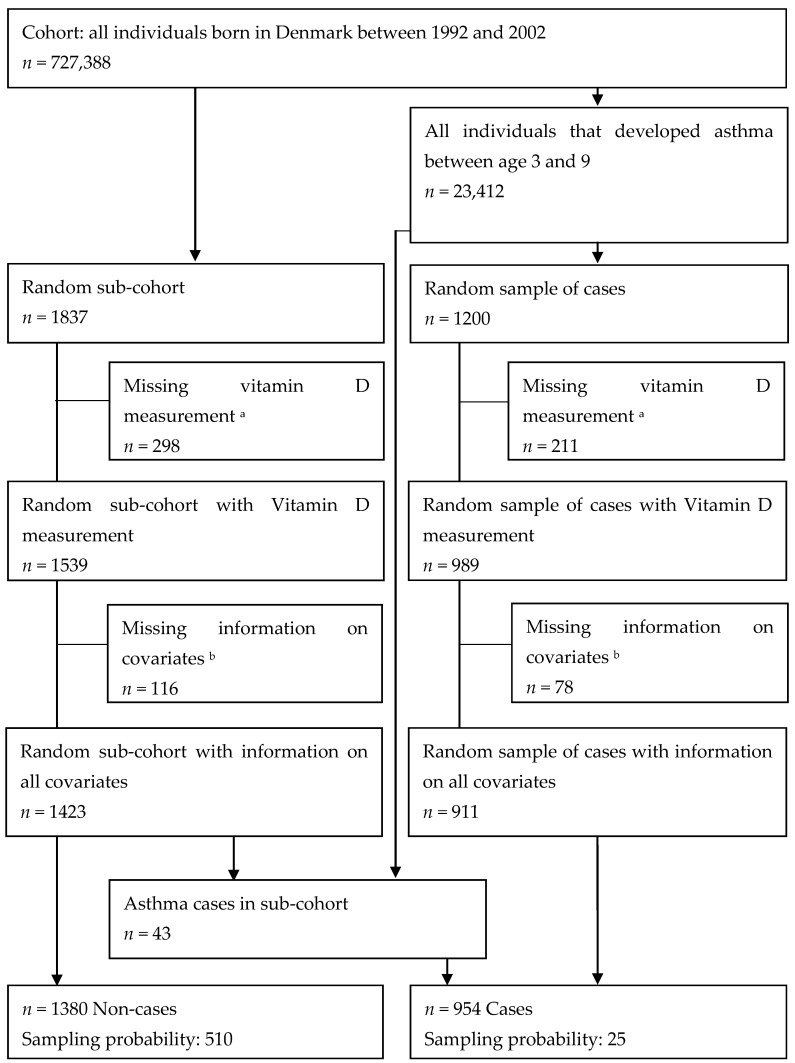
Flowchart of the study population. ^a^ Dried blood spot samples (DBSS) not found, analyses failed or insufficient material for analysis. ^b^ Information on all covariates not available in the registers.

We identified all individuals born in Denmark in 1992–2002 using DCRS. Using the CPR number, we followed individuals in DNPR from age 3–9 years to identify their first asthma diagnosis. We disregarded diagnosis before age 3 due to uncertainties with diagnosis in this age group, as many children suffer from wheezing, bronchitis, or other respiratory symptoms in the first years of life without ever developing asthma [26]. We ended the follow-up at age 9, before puberty, when there is a shift in asthma phenotype from childhood asthma (more prevalent among boys) to adult asthma (more prevalent among women) [4]. Asthma diagnoses covered ICD-8 code 493 and ICD-10 codes J45-6 among inpatients or outpatients as a primary and/or secondary diagnosis. Among all born in Denmark from 1992–2002 (*n* = 727,388), we selected a random sub-cohort of 1837 individuals, and from all asthma cases (*n* = 23,412) we selected 1200 random cases. Their CPR numbers were used to collect DBSSs for vitamin D analysis; 2528 (83%) individuals had DBSS with sufficient material for analysis. We conducted a complete case analysis, excluding individuals with information missing on any covariate. Thus, the final sample consisted of 911 cases and 1423 individuals from the sub-cohort (Figure 1).

### 2.3. Assessment of Vitamin D Status

Vitamin D status was assessed by measuring 25(OH)D_2_ and 25(OH)D_3_ in 3.2 mm punches from DBSSs, at SSI research laboratory. The laboratory participates in the Vitamin D External Quality Assessment Scheme with the equivalent serum analysis method [27], as there are currently no quality assurance programs for 25(OH)D measures in DBSSs. The researchers analyzing the samples were blinded for the outcome and season of birth. Samples were prepared and analyzed using a modified version of a liquid chromatography tandem-mass spectrometry (LC-MS) method [24]. The modification included calibrations and controls produced by using commercially available PerkinElmer MSMS Vitamin D kit (PerkinElmer, Waltham, MA, USA), and on-line two-dimensional step in the extraction procedure. The coefficient variability for intra-assay and inter-assay for 25(OH)D_3_ was 7–12% and 7–20%, respectively, and for 25(OH)D_2_ 4–8% and 9–18%, respectively. For all measured concentrations there was acceptable precision. The lower limit of quantification (LLOQ) was 4 nmol/L for 25(OH)D_3_ and 3 nmol/L for 25(OH)D_2_. We excluded measures of 25(OH)D_2_ from the analysis since 94% of the measures were below LLOQ. All measures of 25(OH)D_3_ were included in the analysis, also those below LLOQ (18%). The 25(OH)D_3_ concentrations are corrected to reflect concentrations equivalent to serum concentrations using the formula: serum 25(OH)D_3_ nmol/L = DBSSs 25(OH)D_3_ nmol/L × 1/[1–0.61], where 0.61 is the hematocrit fraction for capillary blood [22].

### 2.4. Covariates

The following covariates were selected a priori: sex (female, male), month of birth (January to December), season of birth (August–January, February–July), birthweight (continuous, grams), gestational age (preterm <37 weeks, term ≥37 weeks), Caesarean section (yes, no), maternal age (continuous, years), maternal ethnicity (European, non-European), maternal highest obtained education (school, high school, university), parity (primiparous, multiparous), maternal smoking during pregnancy (yes, no), parental asthma (yes, no). Information on child’s sex and month of birth was obtained from DCRS [19]. Birthweight, term of birth, parity, caesarean section, maternal age, and smoking were retrieved from the Danish Medical Birth Register [25]. Presence of parental asthma diagnosis, defined as among the children, was recorded from DNPR [20], and maternal education and ethnicity from Statistics Denmark.

### 2.5. Statistical Analyses

Characteristics of the study population stratified by case and sub-cohort are presented as number (n) and percentages (%) for categorical variables and mean and standard deviation (SD) or median and interquartile range (IQR) for continuous variables. 

Unlike the classic case-cohort design, where all cases are included in the analysis [28], we included a random sample of asthma cases. Therefore, we conducted a weighted Cox regression analysis where both cases and non-cases were weighted with their inverse sampling fractions [29], (Figure 1). Robust standard errors were used to account for extra variance due to sampling and upweighting. Using the Cox proportional hazard model with age as the underlying time variable, we assessed the hazard of first asthma diagnoses between ages 3 and 9 years by quintiles of 25(OH)D_3_, to capture a potential nonlinear relationship, using the first quintile as reference. The results are presented as hazard ratio (HR) and 95% confidence interval (CI). In adjusted model, we adjusted for potential confounders identified a priori (Supplementary Figure S1). In addition, we conducted restricted cubic spline analysis with 3 knots at 10th, 50th and 90th percentile. 

We checked the difference between individuals included in the analysis and excluded due to missing data on covariates using chi-square test and t-test. We did not adjust for smoking in the main analyses due to large percentage of missing data, but ran a sensitivity analysis adjusting for smoking. We further conducted a stratified analysis by sex and season of birth, and tested sex and season interactions with 25(OH)D_3_ quintiles using Wald tests. We also conducted analysis, excluding all children of non-European mothers, to see if the association was modified by ethnicity, and children whose parents have asthma, to see if it was modified by genetic predisposition for asthma. Furthermore, we excluded siblings and individuals with missing information about siblings (*n* = 50, 2.1%) to see if the potential violation of the independency assumption affected the standard error of our estimates. Finally, we ran sensitivity analyses adjusting for region of birth, hypothesizing that hospital admission due to asthma might vary by region. 

All statistical analyses were performed using Stata version 15 (StataCorp. 2017. Stata Statistical Software: Release 15. College Station, TX, USA, www.stata.com). The statistical tests were two sided at a 5% significance level.

### 2.6. Ethical Considerations

Permission to conduct the study was granted by the Ethical Committee of the Capital Region of Denmark (J. no.: H-3-2011-126). The steering committee for scientific use of the Biological Specimen Bank for Neonatal Screening granted permission to access and analyze the DBSSs. Permission to use register data was granted by the Danish Health Data Authority and Statistics Denmark. The Danish Data Protection Agency provided permission to process data (J.no. 2012-41-1156). The study is registered at www.clinicaltrials.gov (NCT03330301).

## 3. Results

Characteristics of the 911 childhood asthma cases and the 1423 children from the random sub-cohort are presented in Table 1. Overall, the 25(OH)D_3_ concentrations in our study were low, ranging from 0–44 nmol/L in the first four quintiles, and in the fifth quintile ranging from 44–111 nmol/L with a mean of 59 nmol/L. The median 25(OH)D_3_ concentration was slightly lower among cases (23 nmol/L, IQR:14–35) than among children from the sub-cohort (25 nmol/L, IQR:14–40). Characteristics of those included (*n* = 2334) in the analysis and those excluded (*n* = 194) from the analysis due to missing information on covariates are presented in Supplementary Table S1. When comparing those included with those excluded, we found that the 25(OH)D_3_ concentration was lower among excluded (19 nmol/L, IQR:10–31) than among included (24 nmol/L, IQR:14–37), and especially among excluded cases (16 nmol/L, IQR:9–27).

**Figure 2:**
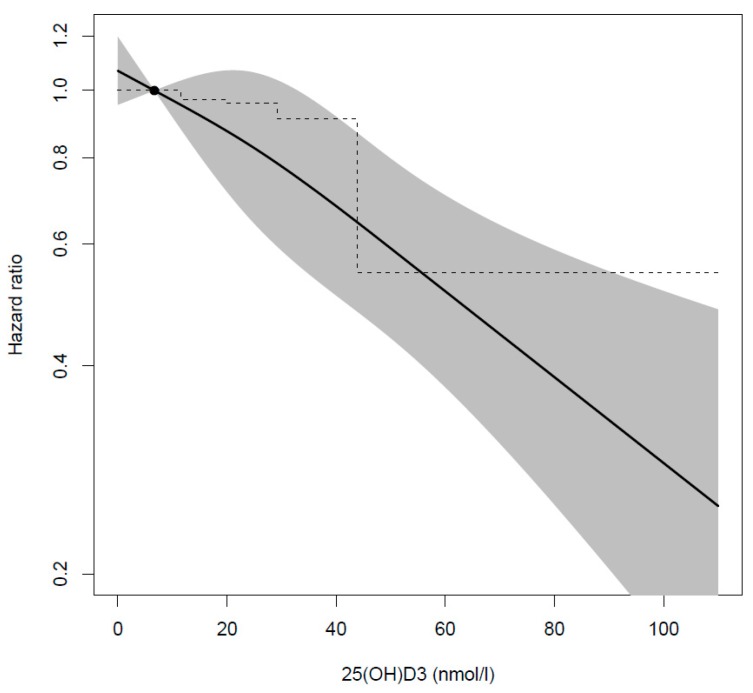
Cubic spline model of the adjusted^a^ hazard ratio^b^ (95% CI) of developing asthma between age 3–9 years and neonatal 25(OH)D_3_ concentrations.

The HR for developing asthma between age 3–9 years was lower for children in the fifth quintile of neonatal 25(OH)D_3_ compared to children in the first quintile, both in the unadjusted (0.61, 95% CI: 0.46–0.80) and adjusted (0.55, 95% CI: 0.39–0.77) analyses, similar estimates were observed in a sensitivity analysis additionally adjusting for maternal smoking (Table 2). There was no significant interaction between categories of 25(OH)D_3_ concentration and sex (all *p* > 0.31) or season of birth (all *p* > 0.10). In analyses stratified by sex and season of birth, similar estimates were found (Table 3); however, the association appeared stronger among those born in August–January (HR 0.51, 95% CI: 0.33–0.79) than those born in February–July (HR 0.66, 95% CI: 0.43–0.99). Adjusting for region of birth, excluding siblings, children who had parents with asthma, and children whose mothers had non-European ethnicity from the analysis, gave similar estimates (Supplementary Table S2). Results from restricted cubic spline analysis showed an inverse close to linear association between neonatal 25(OH)D_3_ concentration and HR of developing asthma (Figure 2).

## 4. Discussion

We observed a lower risk of asthma among children age 3–9 years with the highest compared to lowest neonatal 25(OH)D_3_ concentrations. These results support the hypothesis that prenatal vitamin D concentrations are important for fetal lung and immune system development and decreases the risk of asthma later in life. Several studies on biological mechanisms linking vitamin D and asthma risk provide support for our findings. The programming effects of vitamin D during fetal development have been observed in murine models, where vitamin D deficiency in utero was associated with increase in pulmonary T-helper 2 (Th2) cells and reduction in regulatory T (Treg) cells [30]. In asthma pathogenesis, Th2 causes airway hyperresponsiveness and remodeling through inflammatory pathways, whereas Treg cells suppress this reaction. Moreover, it has been shown that maternal vitamin D deficiency is associated with altered protein expression in the offspring’s lungs [31]. This may alter lung structure and function by altered pulmonary surface and collagen synthesis. In a human clinical study, offspring to mothers supplemented with 4400 IU/d compared to 400 IU/d vitamin D during pregnancy had enhanced proinflammatory cytokine production. Stronger neonatal cytokine responses, especially IFN-γ, are associated with reduced respiratory tract infection and childhood asthma, suggesting that vitamin D strengthens immune responses, and thereby decreases risk of asthma [32].

Similarly to the results from our study two observational studies measuring pregnancy 25(OH)D found an inverse association between 25(OH)D and offspring asthma [33,34]. One found increased risk of asthma at age 6 years, among those with ≤50 nmol/L compared with >50 nmol/L, among boys only [33]; the other found lower risk of offspring asthma at age 4 years among those with maternal 25(OH)D before delivery between 50 and 75 nmol/L compared to <50 nmol/L [34]. However, the majority of previous observational studies measuring 25(OH)D at birth [15,16,35,36,37,38] or at different times during pregnancy [16,18,38,39,40], to examine the association between prenatal vitamin D status and childhood asthma, found no association. It is possible that the studies lacked power to detect a risk difference as the studies had relatively small sample sizes and large confidence intervals. Chawes et al. [15] compared risk of asthma among those that had cord blood 25(OH)D concentrations <50 nmol/L (*n* = 136) to those who had concentrations >75 nmol/L (*n* = 39) and found an increased risk of asthma (OR 1.60); however, not statistically significant (95% CI: 0.49–5.22). One study reported increased risk of offspring asthma at age 9 years among those with 25(OH)D >75 compared to <30 nmol/L in late pregnancy [17]. This is opposite to what we find, where those with 43–111 nmol/L have the lowest risk of developing asthma, also in our spline analyses, the higher the 25(OH)D_3_ status the lower the risk of asthma. However, this study had a very small sample size (19 cases). Furthermore, the inconsistencies between previous studies might be due to differences in 25(OH)D concentrations or methodological differences, such as 25(OH)D measurement method and timing, definition of asthma (e.g., doctor diagnosis or parent reported), age at diagnosis, study design, or statistical analysis applied. Furthermore, the studies were conducted in different populations, at different time points, and varied in follow-up time. 

As in many previous studies, we used 25(OH)D concentrations measured at birth [15,35,36,37,38]. However, our study is the only using neonatal capillary DBSSs, other analyzed serum samples from cord blood. The difference in 25(OH)D concentration between capillary blood and serum sample has been accounted for using the hematocrit fraction for capillary blood. However, our samples were collected up to a week after birth, and it is possible that 25(OH)D concentration might have decreased slightly during this week after birth as exclusive breastfeeding is a predictor of vitamin D deficiency among infants [41]; and in Denmark, <5% of infants are not being breastfed at the time of hospital discharge [42]. However, the tracking of 25(OH)D during pregnancy was previously shown to be moderate [43] and half-life of 25(OH)D is 2–3 weeks; furthermore, vitamin D supplementation for infants is recommended after age 2 weeks; therefore not affecting neonatal 25(OH)D concentrations [44]. Hence, we can be assured that our measurements are a good indicator of the vitamin D concentration in the last month of pregnancy. 

Previous studies defined asthma based on parent reporting and/or physical examination [15,16,17,33,34,35,36,37,38], whereas we have diagnoses in hospital setting. As asthma is most often diagnosed by general practitioners, we probably captured severe or difficult-to-treat asthma cases. The association observed in our study could therefore be specific for this phenotype. Only one previous study conducted longitudinal analysis following children from age 0 to 7 years [15], other studies examined ever diagnosed or current asthma at age ranging from 3 to 7.5 years [16,17,33,34,35,36,37,38]. We started follow-up at age 3 years as asthma is difficult to diagnose before. By constricting the follow-up to age 3–9 years, we can be confident that the child has asthma, and we have complete follow-up for the phenotype of interest: childhood asthma. Finally, compared to previous studies, ours had the largest number of cases included, and is the only study comparing quintiles of vitamin D. Most previous studies compared specific cut-offs, e.g., <50 nmol/L; however, these cut-offs are based on bone-specific outcomes. Sufficient 25(OH)D concentrations for optimal lung and immune system development are unknown. Morales et al. (2012) compared quartiles of 25(OH)D [18]; however, their 25(OH)D concentrations were substantially higher. The first quartile, had concentrations of <54 nmol/L, whereas in our study the first quintile had concentrations of <12 nmol/L. It is possible that the 25(OH)D concentration among the majority of the mothers in the study by Morales et al. (2012) were sufficient for optimal lung and immune system development and, therefore, no effect was observed. Whereas in our study the concentrations in the first quintile might have been insufficient. 

Results from joint analysis of two RCTs [12,13] examining association between prenatal vitamin D supplementation and risk of asthma and recurrent wheeze at age 3 years revealed reduced risk OR 0.74 (95% CI: 0.57–0.96) [14]; however, long-term follow-up at age 6 years showed no association OR 1.21 (95% CI: 0.63–2.32) [45], and interval-censored HR 1.12 (*p* = 0.25) [46]. We found a reduced risk of asthma among children throughout the ages 3–9 years. This difference could be due to methodological aspects such as difference in age intervals and definition of asthma or shortcomings of the case-cohort design. However, it is also possible that the lack of significant results at age 6 years depends on lack of power, as acknowledge in both of the RCTs.

The strength of our study is that we have a large sample of children randomly selected from the entire population of children born in Denmark in 1992–2002 with a measured biomarker of vitamin D concentrations. Result from our study can therefore be generalized to similar populations, such as other western countries. Use of DBSSs instead of plasma or sera has been shown to be an accurate, valid, and reliable alternative to measuring 25(OH)D concentrations [47]. Furthermore, we used high quality register data [48], where asthma diagnoses have been validated against medical records [49], and we adjusted for several potential confounders available in the Danish registers. However, we cannot exclude residual confounding from unknown or unmeasured confounders, e.g., maternal obesity. Obese individuals have greater risk of vitamin D deficiency [50] and their offspring have greater risk of developing asthma [51]. Nevertheless, it is unlikely that the association is explained by maternal obesity, as the prevalence of obesity in Denmark was only 11.8% [52]. Misclassification of cases cannot be excluded; however, the approach we used to identify cases has been validated against medical records and revealed that sensitivity was 0.90, specificity 0.99, positive predictive value 0.85, and negative predictive value 0.99 [49]. Therefore, we consider the definition of cases using the DNPR to be valid. Furthermore, our results are robust and adjusting for potential confounders strengthens the association. We have no reason to believe that loss-to-follow-up (missing 25(OH)D measurements and excluded due to missing covariates) is affected by both exposure and outcome causing bias by selection. Additionally, 25(OH)D_3_ concentrations were lower among excluded cases than among included cases, and among excluded sub-cohort subject compared to included sub-cohort subjects, although to a lesser extent among the sub-cohort. The exclusion of these cases has attenuated the observed association. Another potential limitation to our study is that 25(OH)D_3_ concentrations were considerably lower than concentrations measured in another study in a similar population with measured 25(OH)D from DBSSs [53], and 18% of the concentrations in our study were below the LLOQ. Therefore, we only report quintiles of 25(OH)D_3_ concentration with arbitrary cut-offs, and not internationally comparable standard cut-offs, which have been recommended [6] as being more clinically relevant. Furthermore, we should be cautious interpreting results from the spline analysis and cut-offs for the quintiles of 25(OH)D_3_ concentrations, as the true concentration might be higher than observed in our analysis. Consequently, it is difficult to compare concentrations in our study to previous studies. The reason for the generally low concentrations observed in our study is most likely due to different assessment methods, such as use of different assay, and the true concentration is most likely higher than we have observed; however, we have no reason to believe that this has influenced the ranking of individuals into quintiles of 25(OH)D_3_ concentrations and, therefore, we have no reason to question the association observed. Thus, our results support results from previous studies indicating that neonatal vitamin D status as a proxy of vitamin D status during the prenatal period is important for normal immune- and lung development.

## 5. Conclusions 

Results from our study show a lower risk of developing asthma among children age 3–9 years within the highest quintile of neonatal 25(OH)D_3_ concentration, suggesting that vitamin D status is important for normal immune- and lung development. Same associations were observed among girls and boys, and among children born in both seasons.

## Figures and Tables

**Table 1:** Baseline characteristics of the study population.

	Asthma Cases	Random Sub-Cohort ^†^	*P*-Value
N	911	1423	
25(OH)D_3_ nmol/L median (Q1–Q3)	23 (14–35)	25 (14–40)	0.16
Sex *n* (%)			<0.000
Girls	328 (36)	682 (48)	
Boys	583 (64)	741 (52)	
Season of birth *n* (%)			0.15
August–January	459 (50)	673 (47)	
February–July	452 (50)	750 (53)	
Preterm *n* (%)			0.02
Yes	73 (8)	84 (6)	
Caesarean section *n* (%)			0.01
Yes	120 (13)	139 (10)	
Birthweight in grams mean (SD)	3490 (640)	3517 (577)	0.56
Size for gestational age *n* (%)			0.56
Small for gestational age	117 (13)	167 (12)	
Normal for gestational age	688 (76)	1074 (76)	
Large for gestational age	106 (12)	182 (13)	
Parity *n* (%)			0.86
Primiparous	390 (43)	604 (43)	
Multiparous	521 (57)	819 (58)	
Maternal age in years mean (SD)	28.8 (5)	29.1 (5)	
Maternal ethnicity *n* (%)			0.95
European	836 (92)	1307 (92)	
Non-European	75 (8)	116 (8)	
Maternal education *n* (%)			0.002
School	274 (30)	342 (24)	
High school	421 (46)	748 (53)	
University	216 (24)	333 (23)	
Maternal smoking *n* (%)			0.04
Yes	223 (25)	297 (21)	
Missing	136 (15)	214 (15)	
Maternal asthma *n* (%)			<0.000
Yes	86 (9)	53 (4)	
Paternal asthma *n* (%)			<0.000
Yes	53 (6)	39 (3)	

^†^ In the randomly selected sub-cohort there are 48 cases.

**Table 2:** Unadjusted and adjusted hazard ratio ^†^ (95% CI) of asthma among Danish children age 3–9 years, according to quintiles of neonatal 25(OH)D_3_ concentrations.

	Unadjusted (*n* = 2334)	Adjusted ^‡^ (*n* = 2334)	Adjusted ^§^ (*n* = 1984)
Quintiles limit, nmol/L			
Q1 (0.0–11.6)	1 (ref)	1 (ref)	1 (ref)
Q2 (11.6–20.0)	0.93 (0.72, 1.20)	0.97 (0.74, 1.28)	0.90 (0.66, 1.23)
Q3 (20.0–29.3)	1.00 (0.78, 1.28)	0.96 (0.72, 1.28)	0.94 (0.68, 1.30)
Q4 (29.3–43.9)	0.97 (0.75, 1.25)	0.91 (0.67, 1.23)	0.82 (0.58, 1.14)
Q5 (43.9–110.8)	0.61 (0.46, 0.80)	0.55 (0.39, 0.77)	0.51 (0.35, 0.75)
Wald test	0.002	0.001	0.003

^†^ Weighted Cox regression analysis. ^‡^ Adjusted for sex, month of birth, birthweight, preterm birth, caesarean section, parity, maternal age, maternal ethnicity, maternal education, maternal asthma, and paternal asthma. ^§^ Adjusted for covariates in model ^‡^ and in addition adjusted for maternal smoking.

**Table 3:** Unadjusted and adjusted hazard ratio ^†^ (95% CI) of asthma among Danish children age 3–9 years according to quintiles of neonatal 25(OH)D_3_ concentrations, stratified by sex and season of birth.

	Unadjusted (*n* = 2334)	Adjusted ^‡^ (*n* = 2334)	Adjusted ^§^ (*n* = 1984)
Quintiles limit, nmol/L			
Girls (*n* = 1010)			
Q1 (0.0–11.6)	1 (ref)	1 (ref)	1 (ref)
Q2 (11.6–20.0)	0.88 (0.59, 1.31)	0.88 (0.57, 1.38)	0.84 (0.51, 1.39)
Q3 (20.0–29.3)	1.07 (0.73, 1.56)	1.11 (0.72, 1.71)	0.96 (0.58, 1.59)
Q4 (29.3–43.9)	0.95 (0.63, 1.42)	0.94 (0.58, 1.54)	0.86 (0.49, 1.50)
Q5 (43.9–110.8)	0.58 (0.37, 0.91)	0.62 (0.36, 1.07)	0.52 (0.28, 0.96)
Boys (*n* = 1324)			
Q1 (0.0–11.6)	1 (ref)	1 (ref)	1 (ref)
Q2 (11.6–20.0)	0.96 (0.69, 1.33)	1.05 (0.73, 1.51)	0.97 (0.65, 1.45)
Q3 (20.0–29.3)	0.97 (0.69, 1.36)	0.93 (0.63, 1.38)	1.02 (0.66, 1.58)
Q4 (29.3–43.9)	0.93 (0.67, 1.29)	0.93 (0.63, 1.37)	0.84 (0.55, 1.29)
Q5 (43.9–110.8)	0.60 (0.42, 0.85)	0.53 (0.34, 0.83)	0.54 (0.33, 0.88)
February–July (*n* = 1302)			
Q1 (0.0–11.6)	1 (ref)	1 (ref)	1 (ref)
Q2 (11.6–20.0)	0.94 (0.66, 1.34)	0.92 (0.63, 1.35)	0.83 (0.53, 1.28)
Q3 (20.0–29.3)	1.06 (0.75, 1.51)	0.98 (0.66, 1.44)	0.98 (0.63, 1.53)
Q4 (29.3–43.9)	1.21 (0.85, 1.72)	1.17 (0.80, 1.72)	1.06 (0.69, 1.63)
Q5 (43.9–110.8)	0.72 (0.49, 1.04)	0.66 (0.43, 0.99)	0.63 (0.40, 1.00)
August–January (*n* = 1226)			
Q1 (0.0–11.6)	1 (ref)	1 (ref)	1 (ref)
Q2 (11.6–20.0)	0.90 (0.63, 1.30)	1.00 (0.67, 1.50)	0.97 (0.63, 1.51)
Q3 (20.0–29.3)	0.92 (0.64, 1.32)	0.97 (0.64, 1.47)	0.91 (0.58, 1.43)
Q4 (29.3–43.9)	0.75 (0.52, 1.09)	0.81 (0.54, 1.21)	0.71 (0.46, 1.11)
Q5 (43.9–110.8)	0.50 (0.34, 0.74)	0.51 (0.33, 0.79)	0.48 (0.30, 0.78)

^†^ Weighted Cox regression analysis. ^‡^ Adjusted for sex, month of birth, birthweight, preterm birth, caesarean section, parity, maternal age, maternal ethnicity, maternal education, maternal asthma, and paternal asthma. ^§^ Adjusted for covariates in model ^‡^ and in addition adjusted for maternal smoking.

## Supplementary Materials

The following are available online at https://www.mdpi.com/2072-6643/12/3/842/s1, Figure S1: Potential confounders identified a priory—Directed Acyclic Graph (DAG), Table S1: Characteristics of individuals included in the analysis and excluded from the analysis due to missing data other covariates, Table S2: Unadjusted and adjusted HR^a^ (95% CI) of asthma among Danish children age 3–9 years, according to quintiles of neonatal 25(OH)D_3_ concentrations.
