# Supplementary material for: Neonatal Vitamin D Status and Risk of Asthma in Childhood: Results from the D-Tect Study
Source: Nutrients. 2020 Mar 21;12(3):842. doi: 10.3390/nu12030842 (PMC7146263; doi:10.3390/nu12030842)
Supplement: Supplementary file 1 [file nutrients-12-00842-s001.zip › Supplementary table 1.docx]

| **Supplementary table 1:** Characteristics of individuals included in the analysis and excluded from the analysis due to missing data other covariates. | | | | | | |  |
| --- | --- | --- | --- | --- | --- | --- | --- |
|  | Included | Excluded | P value | Excluded  cases | Excluded  sub-cohort | P value | |
| N | 2,334 | 194 |  | 78 | 116 |  | |
| 25(OH)D_3_ nmol/L median (Q1-Q3) | 24.0 (13.6-37.3) | 19.0 (9.5-31.0) | **0.001** | 16.4 (8.7-27.4) | 21.2 (10.4-33.7) | **0.02** | |
| Sex n (%) |  |  | 0.3 |  |  | 0.5 | |
| Girls | 1010 (43.3) | 91 (46.9) |  | 34 (43.6) | 57 (50.9) |  | |
| Boys | 1324 (56.7) | 103 (53.1) |  | 44 (56.4) | 59 (49.1) |  | |
| Season of birth n (%) |  |  | 1 (0.99) |  |  | 1 (0.95) | |
| August-January | 1132 (45.8) | 94 (48.5) |  | 38 (48.7) | 56 (48.3) |  | |
| February-July | 1202 (51.5) | 100 (51.5) |  | 40 (51.3) | 60 (51.7) |  | |
| Preterm n (%) |  |  | 0.7 |  |  | 0.5 | |
| Yes | 151 (6.5) | 9 (7.4) |  | 3 (5.6) | 6 (9.0) |  | |
| No | 2183 (93.5) | 112 (92.6) |  | 51 (94.4) | 61 (91.0) |  | |
| Caesarean section n (%) |  |  | 0.4 |  |  | 0.06 | |
| Yes | 259 (11.1) | 21 (13.4) |  | 13 (19.4) | 8 (8.9) |  | |
| No | 2075 (88.9) | 136 (86.6) |  | 54 (80.6) | 82 (91.1) |  | |
| Birthweight in grams mean (SD) | 3506 (602) | 3359 (638) | **0.004** | 3345 (614) | 3372 (662) | 0.8 | |
| Parity n (%) |  |  | **0.03** |  |  | 0.4 | |
| Primiparous | 994 (42.6) | 73 (52.1) |  | 35 (56.5) | 38 (48.7) |  | |
| Multiparous | 1340 (57.4) | 67 (47.9) |  | 27 (43.5) | 40 (51.3) |  | |
| Maternal age in years mean (SD) | 29.0 (4.8) | 28.4 (5.3) | 0.1 | 27.9 (5.8) | 28.8 (4.8) | 0.3 | |
| Maternal ethnicity n (%) |  |  | **<0.000** |  |  | 0.8 | |
| European | 2143 (91.8) | 123 (68.0) |  | 52 (66.7) | 71 (68.9) |  | |
| Non- European | 191 (8.2) | 58 (32.0) |  | 26 (33.3) | 32 (31.1) |  | |
| Maternal education n (%) |  |  | 0.2 |  |  | 0.9 | |
| School | 616 (26.4) | 33 (30.3) |  | 14 (31.8) | 19 (29.2) |  | |
| Highschool | 1169 (50.1) | 45 (41.3) |  | 17 (38.6) | 28 (43.1) |  | |
| University | 549 (23.5) | 31 (28.4) |  | 13 (29.6) | 18 (27.7) |  | |
| Maternal smoking n (%) |  |  | 0.4 |  |  | 1 (0.96) | |
| No | 1464 (73.8) | 90 (77.6) |  | 41 (77.4) | 49 (77.8) |  | |
| Yes | 520 (26.2) | 26 (22.4) |  | 12 (22.6) | 14 (22.2) |  | |
| Maternal asthma n (%) |  |  | 0.3 |  |  | **<0.000** | |
| No | 2195 (94.0) | 179 (92.3) |  | 65 (83.3) | 114 (98.3) |  | |
| Yes | 139 (6.0) | 15 (7.7) |  | 13 (16.7) | 2 (1.7) |  | |
| Paternal asthma n (%) |  |  | 0.6 |  |  | 0.6 | |
| No | 2242 (96.1) | 188 (96.9) |  | 75 (96.1) | 113 (97.4) |  | |
| Yes | 92 (3.9) | 6 (3.1) |  | 3 (3.9) | 3 (2.6) |  | |
