# Supplementary material for: Neonatal Vitamin D Status and Risk of Asthma in Childhood: Results from the D-Tect Study
Source: Nutrients. 2020 Mar 21;12(3):842. doi: 10.3390/nu12030842 (PMC7146263; doi:10.3390/nu12030842)
Supplement: Supplementary file 1 [file nutrients-12-00842-s001.zip › Supplementary table 2.docx]

| **Supplementary Table 2.** Unadjusted and adjusted HR^a^ (95% CI) of asthma among Danish children age 3-9 years according to quintiles of neonatal 25(OH)D_3_ concentrations. | | | | |
| --- | --- | --- | --- | --- |
|  | Unadjusted | Adjusted^b^ | Adjusted^c^ | Adjusted^d^ |
| Quintiles limit, nmol/l |  |  |  |  |
| Q1 (0.0-11.6) |  |  |  | 1 (ref) |
| Q2 (11.6-20.0) |  |  |  | 0.96 (0.72, 1.26) |
| Q3 (20.0-29.3) |  |  |  | 0.94 (0.70, 1.27) |
| Q4 (29.3-43.9) |  |  |  | 0,91 (0.67, 1.23) |
| Q5 (43.9-110.8) |  |  |  | **0.54 (0.39, 0.77)** |
| Only western^e^ (n=2143) |  |  |  |  |
| Q1 (0.0-11.6) | 1 (ref) | 1 (ref) | 1 (ref) | 1 (ref) |
| Q2 (11.6-20.0) | 0.87 (0.67, 1.15) | 0.91 (0.68, 1.23) | 0.86 (0.62, 1.20) | 0.90 (0.66, 1.21) |
| Q3 (20.0-29.3) | 0.98 (0.75, 1.28) | 0.97 (0.71, 1.32) | 0.93 (0.66, 1.31) | 0.95 (0.69, 1.29) |
| Q4 (29.3-43.9) | 0.94 (0.72, 1.24) | 0.92 (0.67, 1.27) | 0.84 (0.59, 1.21) | 0.92 (0.67, 1.27) |
| Q5 (43.9-110.8) | **0.61 (0.45, 0.81)** | **0.58 (0.40, 0.83)** | **0.55 (0.37, 0.83)** | **0.57 (0.40, 0.82)** |
| No parental asthma^f^ (n=2106) |  |  |  |  |
| Q1 (0.0-11.6) | 1 (ref) | 1 (ref) | 1 (ref) | 1 (ref) |
| Q2 (11.6-20.0) | 0.82 (0.63, 1.08) | 0.80 (0.60, 1.07) | 0.74 (0.54, 1.02) | 0.78 (0.58, 1.05) |
| Q3 (20.0-29.3) | 0.96 (0.74, 1.26) | 0.93 (0.69, 1.26) | 0.85 (0.61, 1.19) | 0.91 (0.67, 1.23) |
| Q4 (29.3-43.9) | 0.94 (0.72, 1.23) | 0.86 (0.63, 1.18) | 0.74 (0.52, 1.05) | 0.86 (0.63, 1.18) |
| Q5 (43.9-110.8) | **0.61 (0.45, 0.81)** | **0.56 (0.39, 0.81)** | **0.49 (0.32, 0.73)** | **0.54 (0.37, 0.79)** |
| No siblings^g^ (n=2284) |  |  |  |  |
| Q1 (0.0-11.6) | 1 (ref) | 1 (ref) | 1 (ref) | 1 (ref) |
| Q2 (11.6-20.0) | 0.91 (0.70, 1.17) | 0.93 (0.71, 1.23) | 0.87 (0.64, 1.19) | 0.92 (0.69, 1.22) |
| Q3 (20.0-29.3) | 0.97 (0.75, 1.25) | 0.92 (0.68, 1.23) | 0.91 (0.66, 1.16) | 0.90 (0.67, 1.21) |
| Q4 (29.3-43.9) | 0.93 (0.72, 1.20) | 0.86 (0.63, 1.16) | 0.77 (0.55, 1.08) | 0.86 (0.63, 1.17) |
| Q5 (43.9-110.8) | **0.61 (0.46, 0.81)** | **0.53 (0.38, 0.76)** | **0.51 (0.35, 0.74)** | **0.53 (0.38, 0.76)** |
| ^a^ Weighted Cox regression analysis. ^b^Adjusted for sex, month of birth, birthweight, preterm birth, caesarean section, parity, maternal age, maternal ethnicity, maternal education, maternal asthma and paternal asthma, ^c^ adjusted for b and smoking, ^d^ adjusted for b and region, ^e^excluding children off mothers have non-western ethnicity, ^f^excluding children of parents with asthma, ^g^excluding all siblings. | | | | |
