# Supplementary figures and images for: Neonatal Vitamin D Status and Risk of Asthma in Childhood: Results from the D-Tect Study
Source: Nutrients. 2020 Mar 21;12(3):842. doi: 10.3390/nu12030842 (PMC7146263; doi:10.3390/nu12030842)

**Supplementary figure 1**. Potential confounders identified a priory - Directed Acyclic Graph (DAG)


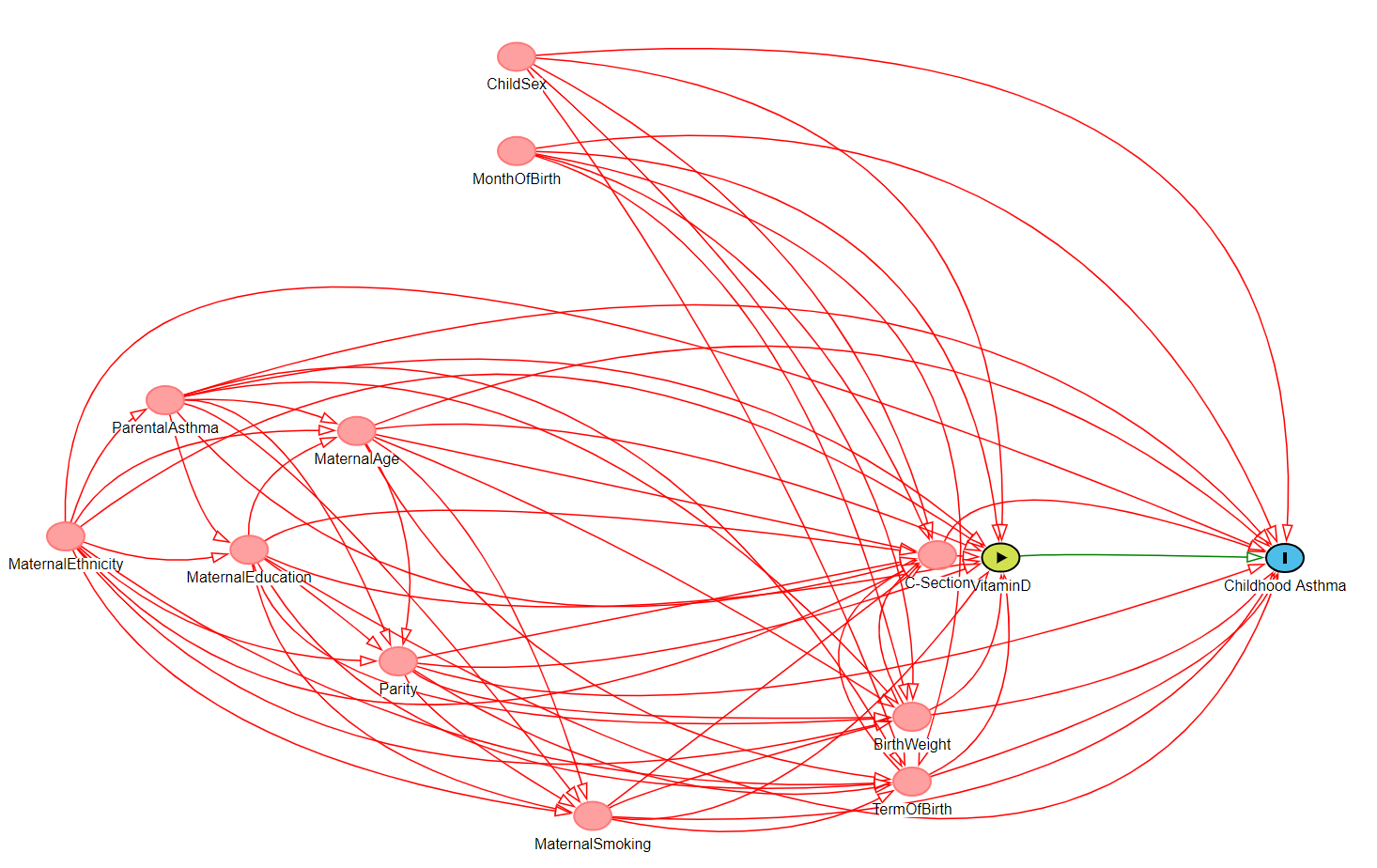

Supplement: Supplementary file 1 [file nutrients-12-00842-s001.zip › Supplementary figure 1.docx]
